# Microarray analysis of differentially expressed microRNAs in myelodysplastic syndromes

**DOI:** 10.1097/MD.0000000000020904

**Published:** 2020-07-02

**Authors:** Chengyao Wan, Jing Wen, Ying Huang, Hongying Li, Wenqi Wu, Qiongni Xie, Xiaolin Liang, Zhongyuan Tang, Weihua Zhao, Peng Cheng, Zhenfang Liu

**Affiliations:** a Department of Hematology, The First Affiliated Hospital of Guangxi Medical University, Nanning, Guangxi; b Department of Hematology, Hainan General Hospital, Haikou, Hainan; c Department of Hematology, The First People's Hospital of Nanning, Nanning, Guangxi, China.

**Keywords:** bioinformatics, microarray, microRNA, myelodysplastic syndromes, pathogenesis

## Abstract

**Background:**

Our study aimed to analyze differential microRNA expression between myelodysplastic syndromes (MDS) and normal bone marrow, and to identify novel microRNAs relevant to MDS pathogenesis.

**Methods:**

MiRNA microarray analysis was used to profile microRNA expression levels in MDS and normal bone marrow. Quantitative real-time polymerase chain reaction was employed to verify differentially expressed microRNAs.

**Results:**

MiRNA microarray analysis showed 96 significantly upregulated (eg, miR-146a-5p, miR-151a-3p, miR-125b-5p) and 198 significantly downregulated (eg, miR-181a-2-3p, miR-124-3p, miR-550a-3p) microRNAs in MDS compared with normal bone marrow. The quantitative real-time polymerase chain reaction confirmed the microarray analysis: expression of six microRNAs (miR-155-5p, miR-146a-5p, miR-151a-3p, miR-221-3p, miR-125b-5p, and miR-10a-5p) was significantly higher in MDS, while 3 microRNAs (miR-181a-2-3p, miR-124-3p, and miR-550a-3p) were significantly downregulated in MDS. Bioinformatics analysis demonstrated that differentially expressed microRNAs might participate in MDS pathogenesis by regulating hematopoiesis, leukocyte migration, leukocyte apoptotic process, and hematopoietic cell lineage.

**Conclusions:**

Our study indicates that differentially expressed microRNAs might play a key role in MDS pathogenesis by regulating potential relevant functional and signaling pathways. Targeting these microRNAs may provide new treatment modalities for MDS.

## 1 Introduction

Myelodysplastic syndromes (MDS) are a group of malignant clonal diseases originating from hematopoietic stem cells, characterized by an abnormal growth of hematopoietic cells, ineffective hematopoiesis, and a high risk of transforming into acute myeloid leukemia (AML).^[1]^ MDS mainly occur in the elderly, as the incidence rate increases with age, and it has high mortality and low cure rates. Gene mutation^[2,3]^ and chromosome abnormality^[4]^ have been reported to be involved in the progression of MDS. However, its molecular pathogenesis and the exact mechanism of transformation into AML have not yet been fully elucidated.

MicroRNA is a type of endogenous non-coding RNA of 19 to 25 nucleotides in length, which is completely or incompletely complementary to the 3’-UTR region of the target gene. Binding of a microRNA regulates gene expression at the post-transcriptional level by the degradation of its target mRNA or inhibition of mRNA translation.^[5]^ Strong evidence suggests that microRNAs play crucial roles in the regulation of hematopoiesis.^[6–8]^ Furthermore, a variety of studies have reported that differentially expressed microRNAs are associated with the transformation of MDS into AML^[9,10]^ and clinical outcomes.^[11–13]^ Ekapun^[14]^ et al reported that DZNep (3-Deazaneplanocin A) could inhibit the expression of let-7b, leading to a decrease in the proportion of cells in the S phase in the MDS-L cell lineage. Recently, there has been a growing interest in microRNA microarray technology to profile microRNAs. MicroRNA expression profiling allows the identification of novel microRNAs associated with MDS pathogenesis.

In the present study, we screened differentially expressed microRNAs in MDS and normal bone marrow using microRNA microarray technology and verified selected microRNAs by quantitative real-time polymerase chain reaction (qRT-PCR), to evaluate novel microRNAs that might be relevant to MDS pathogenesis.

## 2 Materials and methods

### 2.1 Patient samples

Bone marrow (BM) was obtained in the department of Hematology, at The First Affiliated Hospital of Guangxi Medical University, Nanning, China, from 2012 to 2015. BM was extracted from patients at the time of diagnosis. MDS was diagnosed based on the WHO Recommended Criteria (2008), and patients were stratified based on the International Prognostic Scoring System. Patient characteristics are displayed in Table 1. Twelve normal bone marrow samples were obtained from healthy volunteers and donors who were free of any neoplastic disease. All the participants had been given informed consent according to the Declaration of Helsinki. The study was approved by the Human Ethics Committees Review Board at Guangxi Medical University, Nanning, China.

**Table 1:**
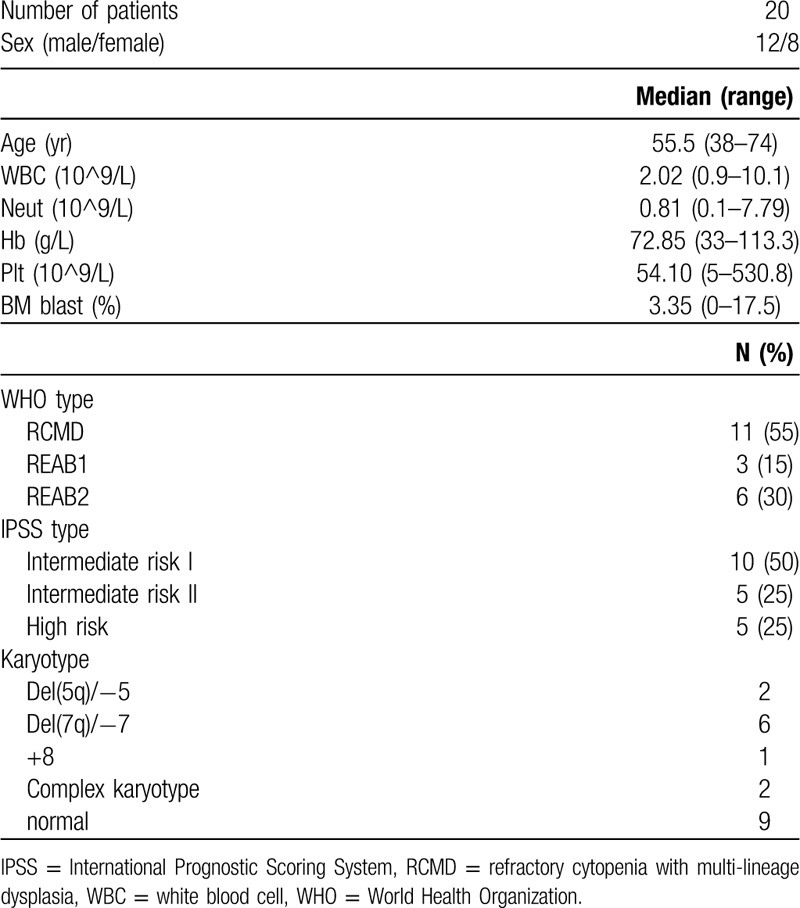
Patient characteristics.

### 2.2 RNA extraction

We separated bone marrow mononuclear cells (BM-MNCs) using density gradient centrifugation. Total RNA was isolated from BM-MNCs of twenty patients and twelve controls using TRIzol reagent (Invitrogen) following the manufacturer's instruction.

### 2.3 miRNA microarray and array data analysis

Total RNA samples were analyzed by the miRCURY LNA Array (v.18.0) (Exiqon).^[15]^ We imported scanned images into GenePix Pro 6.0 soft (Axon) for grid alignment and data extraction. Replicated microRNAs were averaged, and we choose microRNAs with intensities ≥ 30 to calculate the normalization factor. The expressed data were normalized using the median normalization analysis, after which a volcano plot was used to identify significantly differentially expressed microRNAs. A heatmap was created to display microRNA expression profiles of the samples. Statistically significant differentially expressed microRNAs were defined as *P* < .05 and |logFC|> 1.

### 2.4 qRT-PCR validation

The identified differentially expressed miRNAs were validated using the SYBR-based qRT-PCR for twelve MDS and six normal bone marrow samples. Tot-al RNA was reverse transcribed to cDNA. qRT-PCR was performed with SYBR-Green (Invitrogen) in a Rotor-Gene 3000 Real-Time PCR machine (Corbett Research, Australia). The levels of microRNAs were normalized using U6 as an endogenous control. Relative microRNA expression levels were calculated by the 2^−△△ct^ method. The primers were as follows: U6 forward: 5’GCTTCGGCAGCACATATACTAAAAT3’ and reverse: 5’CGCTTCACGAATTTGCGTGTCAT3’; miR-10a-5p forward: 5’GGGTACCCTGTAGATCCGA3’and reverse: 5’CAGTGCGTGTCGTGGAGT3’; miR-146a-5p forward: 5’GGGTGAGAACTGAATTCC3’ and reverse: 5’TGCGTGTCGTGGAGTC3’; miR-125b-5p forward: 5’GCTCCCTGAGACCCTAAC3’ and reverse: 5’GTGCGTGTCGTGGAGTCG3’; miR-221-3p forward: 5’GGGAAGCTACATTGTCTGC3’ and reverse: 5’CAGTGCGTGTCGTGGAGT3’; miR-155-5p forward: 5’GGGGTAATGCTAATCGTGA3’ and reverse: 5’CAGTGCGTGTCGTGGAG3’; miR-151a-3p forward: 5’GGGGCACTAGACTGAAGCTCC3’ and reverse: 5’GTGCGTGTCGTGGAGTCG3’; miR-222-3p forward: 5’GGGGAGCTACATCTGGCT3’ and reverse: 5’TGCGTGTCGTGGAGTC3’; miR-124-3p forward: 5’GGGTAAGGCACGCGGT3’ and reverse: 5’GTGCGTGTCGTGGAGTCG3’; miR-181a-2-3p forward: 5’GGACCACTGACCGTTGAC3’ and reverse: 5’CAGTGCGTGTCGTGGAG3’; miR-550-3p forward: 5’ GGGGTGTCTTACTCCCTCAG3’ and reverse: 5’CAGTGCGTGTCGTGGAGT3’.

### 2.5 Screening for differentially expressed genes (DEGs)

We downloaded the GEO Series (GSE) 114869 and GSE107400 datasets from the expression omnibus database (GEO) database. The platform for GSE114869 is GPL17586, [HTA-2_0] Affymetrix Human Transcriptome Array 2.0 [transcript (gene) version], which includes 300 MDS BM-MNC samples and 20 normal BM-MNC samples. The platform for GSE107400 is GPL17585, [HTA-2_0] Affymetrix Human Transcriptome Array 2.0 [probe set (exon) version], which includes 176 MDS BM-MNC samples and 20 normal BM-MNC samples. Analysis Power Tools (APT)-Release 2.10.2.2 (http://www.affymetrix.com/support/developer/powertools/changelog/) was employed to assess GSE114869 and GSE107400 RAW datasets. We used background correction, quantile normalization, probe summarization, and log2-transformation to create a robust multi-array average (RMA), a log-transformed method. We adjusted the original p-values using the Benjamini-Hochberg method. Statistically significant DEGs were indicated as *P* < .05 and |logFC|> 1. Additionally, common DEGs of the 2 datasets were screened using Venny 2.0.2.

### 2.6 Bioinformatics analysis

The TargetScan database, miRandan database, and miRDB database were assessed to predict target genes of differently expressed microRNAs. Subsequently, these predicted target genes were integrated with the identified common DEGs of the GSE114869 and GSE107400 datasets to obtain potential target genes of validated microRNAs. Then these identified potential targets underwent gene ontology (GO) classification and Kyoto Encyclopedia of Genes and Genomes (KEGG) analysis for functional and signaling pathway analysis. P < 0.05 indicated statistical significance. Additionally, the String database (Available at: http://string-db.org), an online tool used for the structural and functional analysis of protein interactions, was designed to construct a PPI network of potential microRNA target genes.

### 2.7 Statistical analysis

The SPSS version 17.0 (SPSS lnc., Chicago, IL) was employed for statistical analysis. The Student *t* test (2-sided) was employed for comparison of 2-group parameters. *P* < .05 was considered statistically significant.

## 3 Results

### 3.1 Study design and analysis

**Figure 1:**
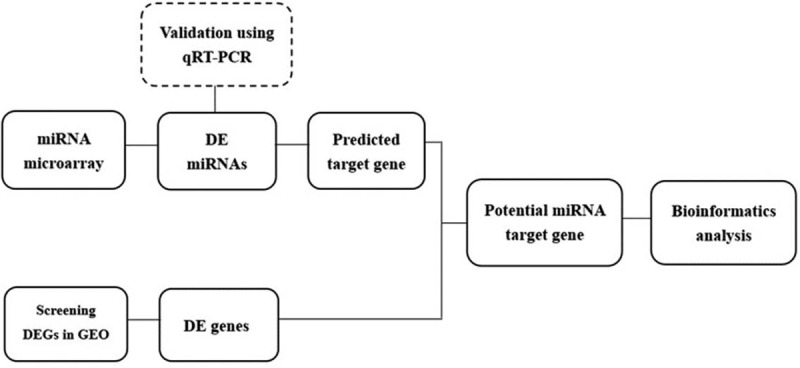
Predicted target gene of differentially expressed microRNAs between MDS and healthy controls were integrated with the DEGs identified from the GEO dataset. Bioinformatics analysis was conducted to identify which pathways were regulated by differentially expressed microRNAs.

Twenty MDS patients and twelve healthy controls were included in the study. From these samples, 8 patients (aged 47 to 73 years, 5 males and 3 females) and 6 healthy controls (aged 46 to 61 years, 3 males, and 3 females) were used for the microarray study. Another twelve MDS patients (aged 38 to 67 years, 7 males and 5 females) and 6 normal controls (aged 41 to 52 years, 4 males and 2 females) were used for qRT-PCR validation. Identified differentially expressed microRNAs were validated by qRT-PCR. Differentially expressed mRNAs were discovered using the GEO dataset. Potential microRNA target genes were identified by a prediction algorithm analysis and exhibited differential expression in the GEO dataset. Subsequently, the potential microRNA target genes were subjected to bioinformatics analysis (Fig. 1).

### 3.2 Identification of differentially expressed microRNAs

Compared with normal bone marrow, a total of 96 statistically upregulated (eg, miR-146a-5p, miR-151a-3p, miR-125b-5p), and 198 statistically downregulated (eg, miR-181a-2-3p, miR-124-3p, and miR-550a-3p) microRNAs were screened based on having a 2-fold change. Differentially expressed microRNAs were presented in a volcano plot and heatmap diagram (Fig. 2 A and Fig. 2 B), and were used in a scatter plot analysis (Fig. 2 C).

**Figure 2:**
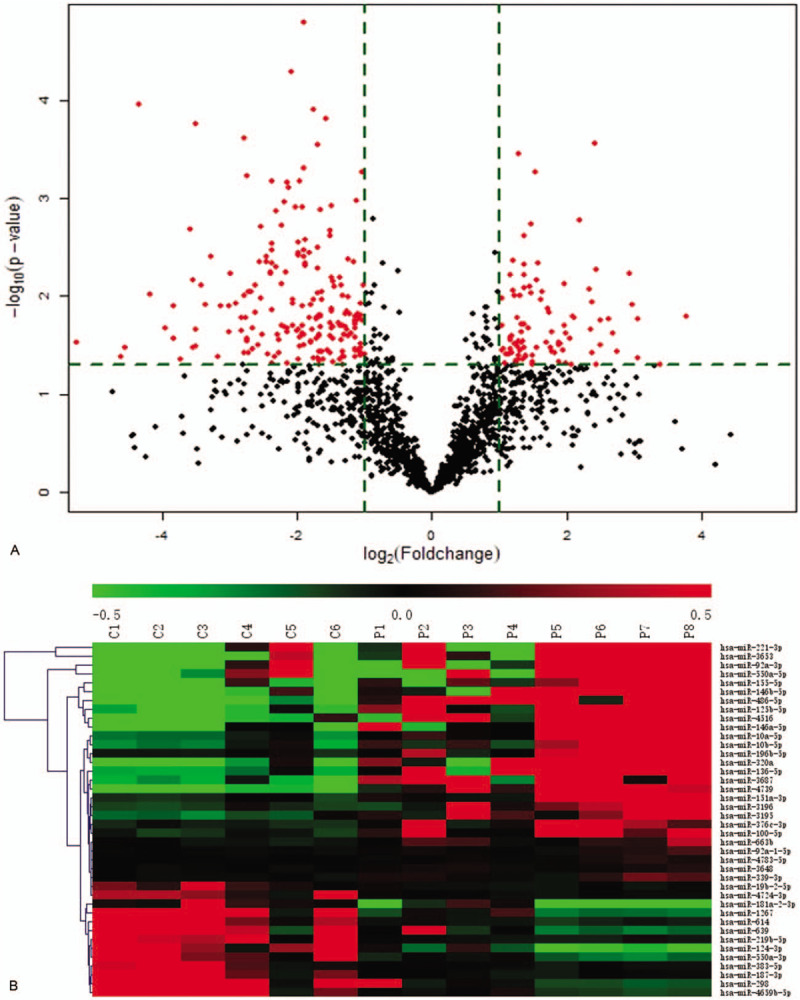
A. Volcano plots of the microarray analysis. Plots show differences between MDS and normal bone marrow. Differentially expressed microRNAs with log|FC| > 1 and P < 0.05 are shown in red. B. MicroRNA expression patterns in MDS compared with normal bone marrow. Each row represents a miRNA and each column represents a sample. C1-C6 represent controls (n = 6), P1-P4 represent MDS patients with intermediate I (n = 4), P5-P8 represent MDS patients with intermediate II and high-risk (n = 4). Red color indicates up-regulation and green color indicates down-regulation. C. Differentially expressed microRNAs between MDS and normal bone marrow in a scatter plot analysis.

**Figure 2 (Continued):**
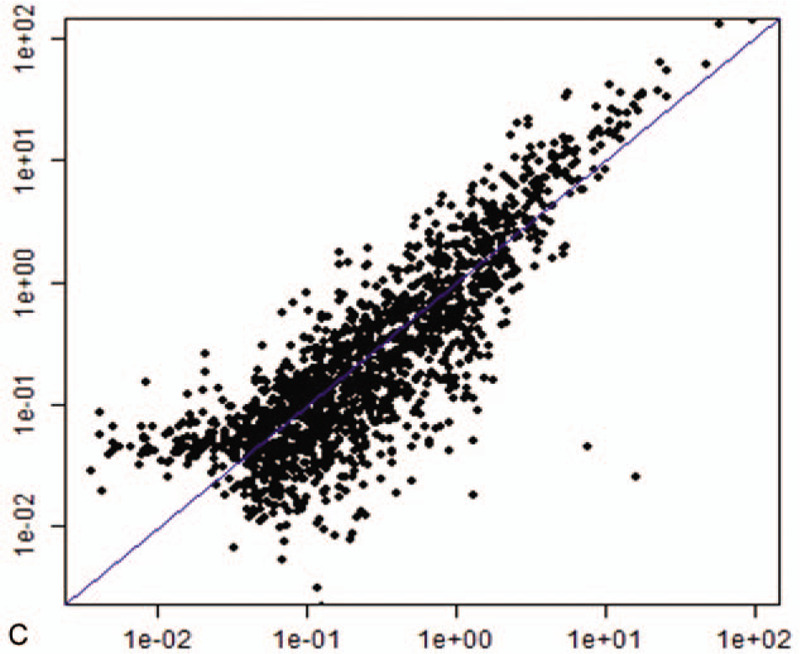
A. Volcano plots of the microarray analysis. Plots show differences between MDS and normal bone marrow. Differentially expressed microRNAs with log|FC| > 1 and P < 0.05 are shown in red. B. MicroRNA expression patterns in MDS compared with normal bone marrow. Each row represents a miRNA and each column represents a sample. C1-C6 represent controls (n = 6), P1-P4 represent MDS patients with intermediate I (n = 4), P5-P8 represent MDS patients with intermediate II and high-risk (n = 4). Red color indicates up-regulation and green color indicates down-regulation. C. Differentially expressed microRNAs between MDS and normal bone marrow in a scatter plot analysis.

### 3.3 qRT-PCR validation for differentially-expressed microRNAs

To confirm the microarray results, we selected the most significantly differentially expressed microRNAs (fold-change>5 and *P* < .05) for qRT-PCR validation, including 7 upregulated microRNAs (miR-155-5p, miR-146a-5p, miR-151a-3p, miR-221-3p, miR-125b-5p, miR-10a-5p, miR-136-5p) and 3 downregulated microRNAs (miR-181a-2-3p, miR-124-3p, miR-550a-3p). 12 MDS patients were divided into a lower risk (low+intermediate I) and a higher risk group (intermediate II+high) based on the International Prognostic Scoring System. Compared to the normal controls, miR-155-5p, miR-146a-5p, miR-151a-3p, miR-221-3p, miR-125b-5p, and miR-10a-5p were significantly elevated and miR-181a-2-3p was significantly decreased in each subgroup of MDS. miR-124-3p and miR-550a-3p were significantly downregulated in the higher risk group of MDS while having an decreased trend in the lower risk group (*P* = .08 and *P* = .10, respectively). Compared to the lower risk group, miR-155-5p, miR-146a-5p, miR-151a-3p, miR-221-3p, miR-125b-5p, and miR-10a-5p were significantly higher and miR-124-3p, miR-181a-2-3p were significantly lower expressed in the higher risk group. However, miR-136-5p had an increased trend in the lower risk group and a decreased trend in the higher risk group (*P* = .10 and *P* = .10, respectively), and the expression of miR-136-5p was significantly lower in the higher risk than in the lower risk group (Fig. 3 ).

**Figure 3:**
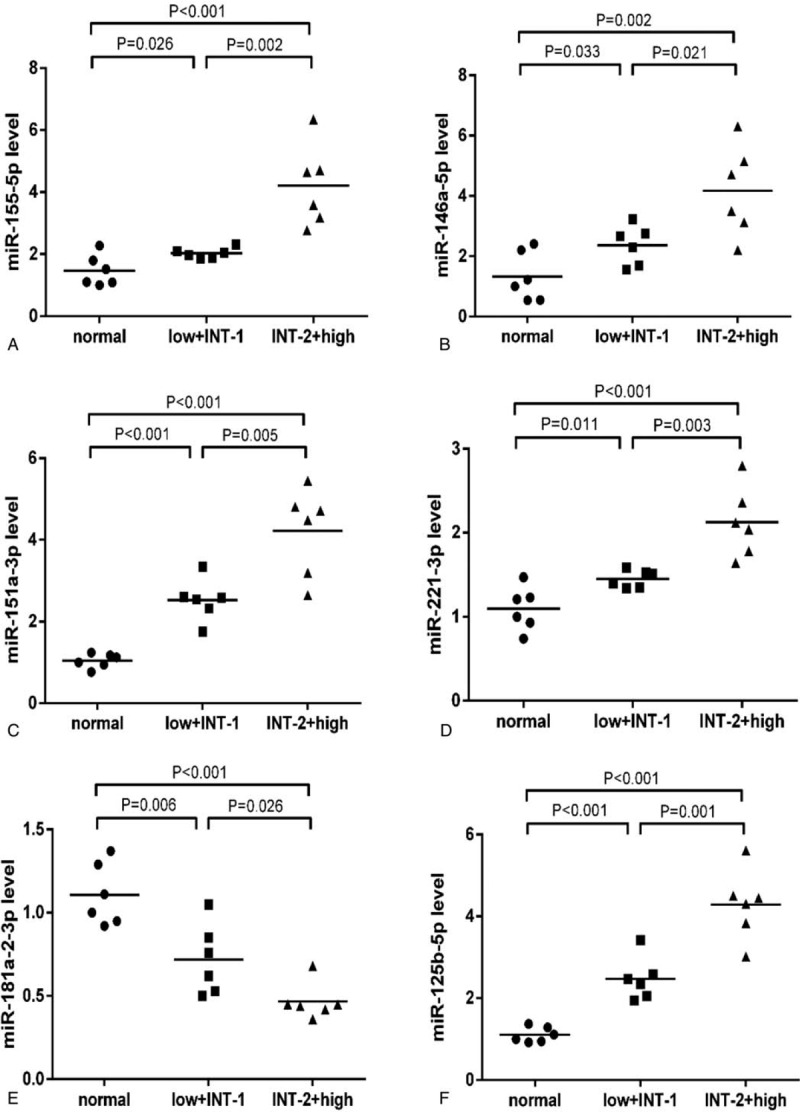
qRT-PCR validation for differentially-expressed microRNAs in each group. a: For miR-155-5p; b: For miR-146a-5p; c: For miR-151a-3p; d: For miR-221-3p; e: For miR-181a-2-3p; f: For miR-125b-5p; g: For miR-124-3p; h: For miR-550a-3p; I: For miR-10a-5p; J: For miR-136-5p. qRT-PCR = quantitative real-time polymerase chain reaction.

**Figure 3 (Continued):**
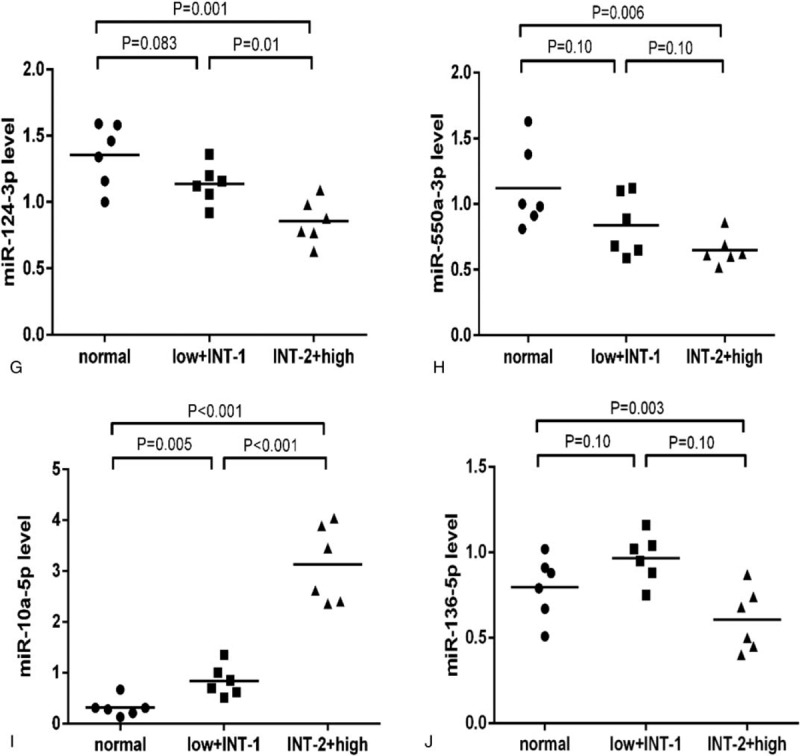
qRT-PCR validation for differentially-expressed microRNAs in each group. a: For miR-155-5p; b: For miR-146a-5p; c: For miR-151a-3p; d: For miR-221-3p; e: For miR-181a-2-3p; f: For miR-125b-5p; g: For miR-124-3p; h: For miR-550a-3p; I: For miR-10a-5p; J: For miR-136-5p. qRT-PCR = quantitative real-time polymerase chain reaction.

### 3.4 Identification of DEGs in MDS

**Figure 4:**
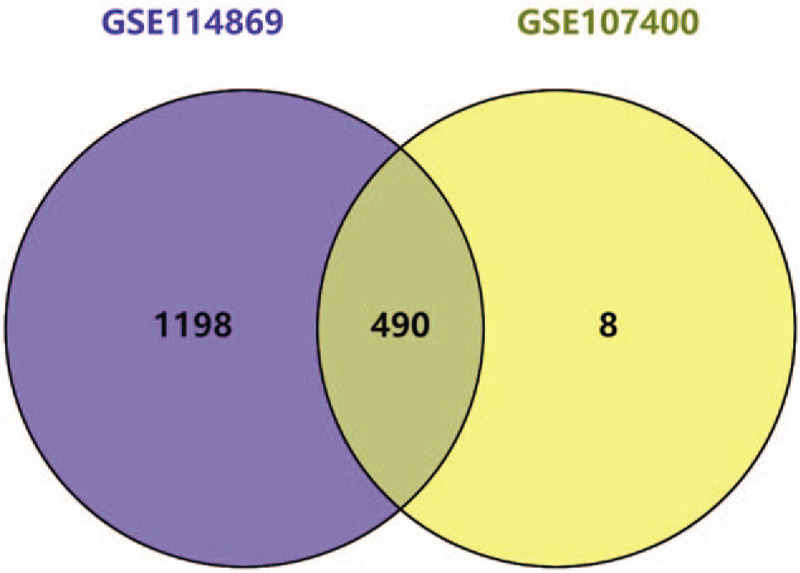
Venn diagram of DEGs. 490 common DEGs were screened out from the two GEO datasets (GSE114869,GSE107400).

A total of 67,528 probes corresponding to 25,875 genes were identified in the GSE114869 and GSE107400 datasets. Statistically significant DEGs were indicated as *P* < .05 and |logFC| > 1. Using Venny 2.0.2, we found 490 common DEGs between MDS and normal bone marrow in the GSE114869 and GSE107400 datasets (Fig. 4). The 490 common DEGs were used as identification criteria for potential microRNA target genes.

### 3.5 Bioinformatics analysis

**Figure 5:**
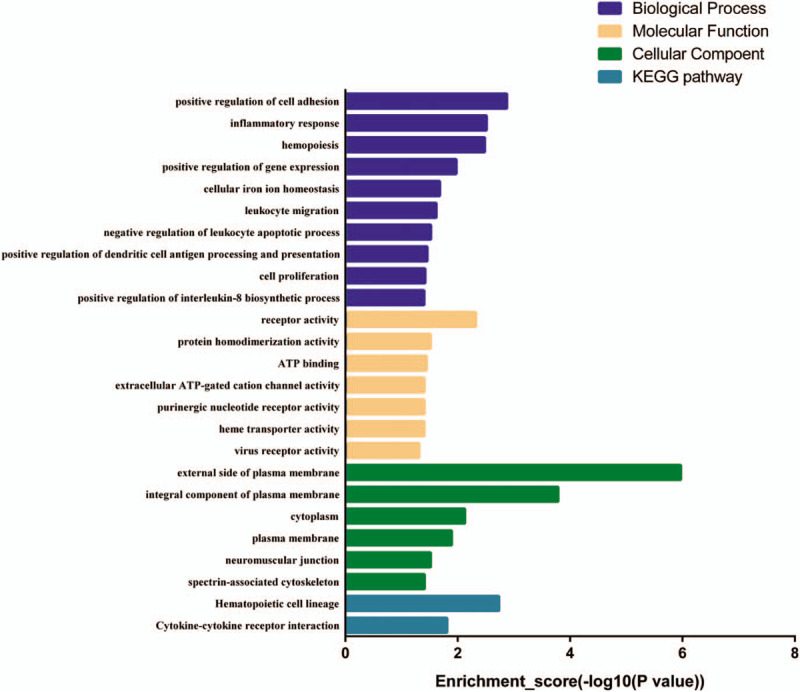
GO classification and KEGG pathway of potential miRNA target genes. KEGG = Kyoto Encyclopedia of Genes and Genomes.

**Figure 6:**
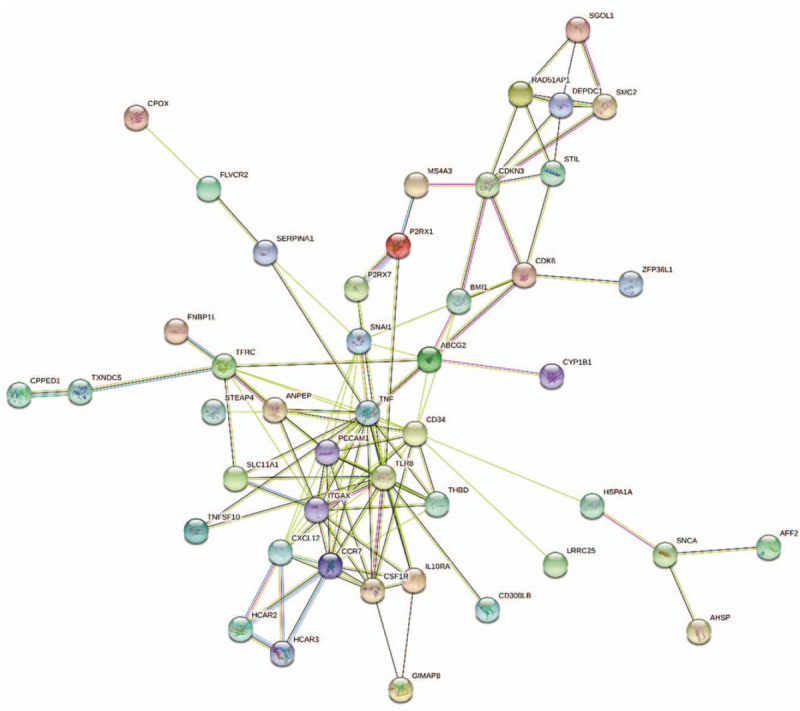
PPI network. Dots represent genes, and lines represent the interactions. PPI = protein protein interaction.

We used the TargetScan, miRandan, and miRDB databases to predict the target genes of the 9 validated microRNAs. To improve the reliability of the predicted target genes, we intersected the predicted target genes with the identified DEGs to obtain the potential microRNA target genes. As a result, 96 potential microRNA target genes were identified (Table 2). To further evaluate the potential implications for these validated microRNAs, GO analysis was conducted to estimate the function of the identified target genes, including biological processes, molecular functions, and cellular components. When assessing biological processes, target genes were classified into 73 categories, including involvement in positive regulation of cell adhesion, inflammatory response, and hemopoiesis. For molecular functions, the results included receptor activity, protein homodimerization activity, and ATP binding. Finally, the cellular components mainly involved the external side of the plasma membrane, the integral component of the plasma membrane, and the cytoplasm (Fig. 5). KEGG pathway analysis revealed that potential microRNA target genes might play roles in hematopoietic cell lineage and cytokine-cytokine receptor interaction (Fig. 5). Additionally, we employed the STRING database (Available at: http://string-db.org) to create PPI networks for the 96 identified potential microRNA target genes. After removing the isolated and partially connected nodes, a complicated network of potential microRNA target genes was constructed (Fig. 6)

**Table 2:**
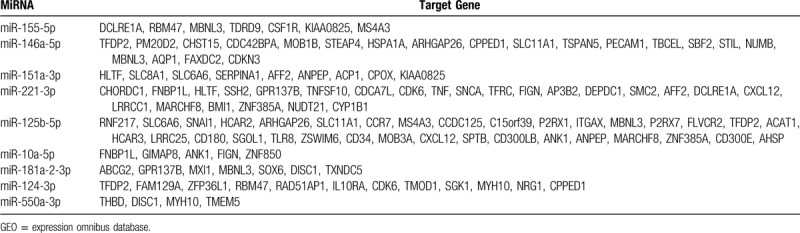
Genes were predicted by the 3 prediction algorithms and showed a differential expression in the 2 GEO datasets.

## 4 Discussion

In this study, we evaluated differentially expressed microRNAs between MDS and normal bone marrow samples using microarray analysis, a powerful technology widely employed to discover genome-wide expression variability of microRNAs. A total of 96 upregulated and 198 downregulated microRNAs were identified in MDS. Among the differentially expressed microRNAs in microarray results, ten selected microRNAs were also detected using qRT-PCR. The expression of 9 microRNAs (eg, miR-146a-5p, miR-151a-3p, miR-125b-5p) was consistent with microarray results, indicating these 9 micro

RNAs might provide a significant contribution to MDS pathogenesis. The disaccord of miR-136-5p expression between microarray identification and qRT-PCR verification might be attributed to the false-positive results of the microarray. Moreover, we will conduct a large sample size for further study.

miR-155 has been demonstrated to be dysregulated in different types of malignancies, such as cervical cancer,^[16]^ breast cancer,^[17]^ colon cancer,^[18]^ gastric cancer,^[19]^ as well as AML.^[20]^ In cervical cancer, miR-155 promotes malignant tumor cell phenotypes through direct targeting of TP53INP1.^[21]^ Additionally, miR-155 is overexpressed in AML and was identified as a potential biomarker for detecting AML.^[22]^ Wang^[23]^ et al. demonstrated that miR-146a can promote cell proliferation and suppresses cell apoptosis via the downregulation of CNTFR in AML and ALL. In another study, Spinello^[24]^ et al found that miR-146a was remarkably elevated in AML and promoted leukemogenesis through targeting of CXCR4. In our study, miR-146a was upregulated with an approximately 2.79-fold change in MDS compared with normal, indicating that miR-146a may have a similar effect in MDS. Additionally, Lee^[25]^ et al demonstrated that miR-221 was markedly overexpressed in AML. In an earlier study, Georgiantas^[26]^ et al reported that miR-221 might serve as a myelopoiesis suppressor by inhibiting molecules involved in myeloid development. Similar to AML, MDS is characterized by myeloid development abnormalities. Therefore, we inferred that the overexpression of miR-221 might promote MDS progression via the inhibition of myeloid development. It has been suggested that miR-125b is also overexpressed and can inhibit myeloid cell differentiation in AML and MDS.^[27]^ Consistent with the previous study, our study found that miR-125b was upregulated with an approximate 2.89-fold change in MDS compared with normal control. A variety of studies have demonstrated that miR-10a is overexpressed and correlates with an adverse prognosis in AML.^[28,29]^ In addition, mir-10a also plays a key role in myeloid differentiation.^[29]^ In our study, microarray results and qRT-PCR validation both revealed that miR-10a was significantly elevated in MDS compared with normal control, suggesting that miR-10a may have a similar effect in MDS. It has been reported that a higher level of miR-181a-2 is correlated with better clinical outcome in patients with AML.^[30]^ Considering the prognostic role of miR-181a-2 in AML, we inferred that the downregulation of miR-181a-2 might contribute to the progression of MDS. Additionally, Wang^[11]^ et al demonstrated that miR-124 is hypermethylated in MDS, and its hypermethylation is significantly correlated with an adverse prognosis. Likewise, we found that the miR-124 expression in MDS was significantly lower than normal control in our study.

In this study, GO analysis showed that differentially expressed microRNAs were involved in biological processes, such as hematopoiesis, leukocyte migration, and negative regulation of leukocyte apoptotic processes, which have been reported to be major contributor to MDS pathogenesis,^[31–33]^ indicating that the differentially expressed microRNAs may participate in the progression of MDS. The KEGG pathway analysis displayed an involvement of the hematopoietic cell lineage. Previous studies have shown that MDS is characterized by the abnormal development of 1 or more hematopoietic cell lineages.^[34–36]^ This may explain how the differentially expressed microRNAs contribute to the progression of MDS. We created a PPI network of the potential miRNA target genes and discovered 45 closely related genes.

Of the microRNAs verified in this study, miR-151a-3p and miR-550–3p have not been evaluated before in hematopoietic malignancies, but they have been demonstrated to play a critical role in other cancers. Latchana^[37]^ et al reported that the expression of miR-151a-3p was significantly decreased in the plasma of metastatic melanoma patients after surgical resection, indicating miR-151a-3p may serve as an oncogene in metastatic melanoma. Similarly, Zhu^[38]^ et al confirmed that miR-151a-3p was markedly elevated in metastatic renal cell carcinoma and might promote carcinogenesis by targeting MCL1. In our study, miR-151a-3p was also markedly elevated in MDS. Further experiments are required to explore the effect of miR-151a-3p in MDS. One study reported that miR-550a-3p was reduced in breast cancer and was associated with inhibition of disease development.^[39]^ In this study, ERK1 and ERK2 were confirmed to be the target genes of miR-550a-3p. Downregulation of miR-550a-3p leads to the upregulation of ERK1 and ERK2. In a previous study, ERK1/2 has been demonstrated to contribute to the transformation of MDS into AML.^[40]^ Therefore, we inferred that the downregulation of miR-550a-3p might promote the progression of MDS by upregulating ERK1/2. The potential mechanisms by which miR-550a-3p is involved with should be evaluated in MDS cell culture models in future studies.

In conclusion, our study revealed 96 significantly upregulated (eg, miR-146a-5p, miR-151a-3p, miR-125b-5p) and 198 significantly downregulated (eg, miR-181a-2-3p, miR-124-3p, miR-550a-3p) microRNAs in MDS compared with normal bone marrow. The PCR results confirmed the microarray analysis: 6 microRNAs (miR-155-5p, miR-146a-5p, miR-151a-3p, miR-221-3p, miR-125b-5p, miR-10a-5p) expressed significantly higher while 3 microRNAs (miR-181a-2-3p,miR-124-3p,miR-550a-3p) exhibited an obviously lower expression in MDS compared with control. The GO classification and KEGG analysis demonstrated that differentially expressed micoRNAs may participate in MDS pathogenesis by regulating hematopoiesis, leukocyte migration, leukocyte apoptotic process, and hematopoietic cell lineage. Further researches are required to explore the exact mechanism for the differentially expressed microRNAs in MDS pathogenesis. Hopefully, our study might provide new strategies for the diagnosis and therapy of MDS.

## Author contributions

**Conceptualization:** Chengyao Wan, Zhenfang Liu.

**Data curation:** Jing Wen, Ying Huang, Hongying Li.

**Formal analysis:** Chengyao Wan.

**Funding acquisition:** Zhenfang Liu.

**Investigation:** Chengyao Wan, Qiongni Xie, Xiaolin Liang.

**Resources:** Wenqi Wu, Zhongyuan Tang.

**Writing – original draft:** Chengyao Wan.

**Writing – review & editing:** Weihua Zhao, Peng Cheng, Zhenfang Liu.
